# Clinical factors associated with safety and efficacy in patients receiving direct oral anticoagulants for non-valvular atrial fibrillation

**DOI:** 10.1038/s41598-020-77174-z

**Published:** 2020-11-19

**Authors:** Hiroshi Yamato, Koichiro Abe, Shun Osumi, Daisuke Yanagisawa, Shinya Kodashima, Yoshinari Asaoka, Kumiko Konno, Ken Kozuma, Takatsugu Yamamoto, Atsushi Tanaka

**Affiliations:** 1 Division of Gastroenterology, Department of Medicine, Teikyo University School of Medicine, 2-11-1 Kaga, Itabashi-ku, Tokyo 173-8605 Japan; 2 Division of Cardiology, Department of Medicine, Teikyo University School of Medicine, 2-11-1 Kaga, Itabashi-ku, Tokyo 173-8605 Japan

**Keywords:** Gastroenterology, Risk factors, Signs and symptoms

## Abstract

Although patients suffering from atrial fibrillation have increased worldwide, detailed information about factors associated with bleeding during direct oral anticoagulant therapy remains insufficient. We studied 1086 patients for whom direct oral anticoagulants were initiated for non-valvular atrial fibrillation between April 2011 and June 2017. Endpoints were clinically relevant bleeding or major adverse cardiac and cerebrovascular events until the end of December 2018. Incidences of bleeding and thrombosis were 4.5 per 100 person-years and 4.7 per 100 person-years, respectively. Most bleeding events represented gastrointestinal bleeding. Multivariate analysis revealed initiation of anticoagulants at ≥ 85 years old as significantly associated with bleeding, particularly gastrointestinal bleeding, but not major cardiac and cerebrovascular events. Other significant factors included chronic kidney disease, low-dose aspirin and nonsteroidal anti-inflammatory drugs. For gastrointestinal bleeding alone, histories of gastrointestinal bleeding and malignancy also showed positive correlations, in addition to the above-mentioned factors. Clinicians should pay greater attention to the risk of gastrointestinal bleeding when considering prescription of anticoagulants to patients ≥ 85 years old with atrial fibrillation.

## Introduction

Bleeding is the most common adverse event in patients taking antithrombotic agents. Since this complication sometimes becomes severe and can worsen the prognosis of the original diseases, adequate management of the risk is clinically essential^1^. Recently, the number of patients with atrial fibrillation (AF) has increased worldwide^2^. As this arrhythmia greatly increases the risk of serious thrombotic events such as cerebral infarction, prophylactic administration of anticoagulants is highly recommended for patients with AF^3^. Hemorrhagic adverse events are also frequent during anticoagulant therapy, so a balance between the risks of thrombosis and bleeding is important^4,5^. Novel direct oral anticoagulants (DOACs) show significant prophylactic effects against thrombosis in AF patients comparable to or better than those of conventional warfarin, while the incidence of hemorrhagic complications seems lower than that with warfarin^6–9^. This feature is one reason why use of DOACs has been increasing over time^10^.

Some risk factors for bleeding in AF patients taking vitamin K antagonists have been identified. Components of the HAS-BLED score include hypertension, abnormal renal/liver function, history of stroke, history of bleeding, labile international normalized ratio, elderly, and drug/alcohol abuse^11–15^. However, the impact of clinical factors on bleeding during DOAC therapy has yet to be fully elucidated, particularly in real-world settings. For example, age is one of the factors significantly affecting the incidence of bleeding in some research, with patients ≥ 75 years old showing a higher risk of bleeding. Meanwhile, very elderly patients (≥ 85 years old) are increasingly encountered in clinical settings, particularly in developed nations where the ratio of this population has increased. As the incidence of thrombosis in AF patients without anticoagulants rises rapidly with age, some researchers have claimed that anticoagulants should be administered even for the very elderly. However, clinicians might want to know which type of bleeding would increase, the severity and frequency, and related background factors. Unfortunately, we do not yet have answers to these questions, and information about the safety of DOACs for very elderly AF patients remains limited^16–19^.

We conducted a retrospective cohort study to clarify the safety and efficacy of DOACs among patients with AF, with a focus on very elderly patients ≥ 85 years old.

## Results

### Baseline characteristics

Table 1 shows the baseline characteristics of study subjects. Median age was 73 years old, and 112 patients (10.3%) were ≥ 85 years old. Two-thirds of subjects were male. Median CHADS2 and CHA2DS2-VASc scores were 2 and 4, respectively. Types of DOAC were as follows: dabigatran, 221 (20.3%); rivaroxaban, 477 (43.9%); apixaban, 322 (29.7%); and edoxaban, 68 (6.3%). In the present study, 752 patients (69.2%) were prescribed at the recommended dose indicated in the guideline^20^. Conversely, 334 patients (30.8%) were treated with dosages inconsistent with recommendations, mostly with underdosing. Only for patients ≥ 85 years old, the proportion receiving the recommended dose was 62.6%, and 35.7% were underdosed. Regarding comorbidities, prevalence was 80% for hypertension, 58% for chronic heart failure (CHF), 4% for severe chronic kidney disease (CKD), and 9% for advanced malignancy. In terms of concomitant medications, low-dose aspirin (LDA) was prescribed in 20.2%, nonsteroidal anti-inflammatory drugs (NSAIDs) in 3.1%, and proton pump inhibitors (PPIs) in 54.6%.

**Table 1:** Baseline characteristics of patients.

Characteristics	Data
Number of patients	1086
Age (year; median (IQR))	73 (66–80)
Very elderly (≥ 85); n (%)	112 (10.3)
Sex (male); n (%)	734 (67.6)
Height (m; median (IQR))	1.63 (1.54–1.69)
Weight (kg; median (IQR))	61.8 (53.0–70.8)
** ≥ **100 kg; n (%)	16 (1.5)
≥ 60 to < 100 kg; n (%)	595 (54.8)
≥ 40 to < 60 kg; n (%)	434 (40.0)
< 40 kg; n (%)	41 (3.8)
CHADS2 score (median (IQR))	2 (2–3)
CHA2DS2-VASc score (median (IQR))	4 (3–5)
eGFR (mL/min/1.73 m^2^; median (IQR))	57.7 (47.8–68.5)
**Direct oral anticoagulant; n (%)**	
Dabigatran	221 (20.3)
Rivaroxaban	477 (43.9)
Apixaban	322 (29.7)
Edoxaban	68 (6.3)
Overdose	29 (2.7)
Standard dose	752 (69.2)
Underdose	305 (28.1)
**Comorbidities; n (%)**	
Hypertension	903 (83.1)
Diabetes mellitus	280 (25.8)
Dyslipidemia	534 (49.7)
Chronic heart failure	634 (58.4)
Ischemic heart disease	273 (25.1)
Cerebrovascular disease	164 (15.1)
Peripheral arterial disease	63 (5.8)
Chronic obstructive pulmonary disease	75 (6.9)
Liver cirrhosis	1 (0.0)
Advanced malignancy	100 (9.2)
End-stage CKD (eGFR < 30 mL/min/1.73 m^2^)	33 (3.9)
History of gastrointestinal bleeding	18 (1.7)
**Medications; n (%)**	
Low-dose aspirin	219 (20.2)
Adenosine 2 phosphate receptor P2Y12 antagonist	142 (13.1)
Nonsteroidal anti-inflammatory drugs	34 (3.1)
Steroids	47 (4.3)
Proton pump inhibitor	593 (54.6)

IQR; interquartile range, eGFR; estimated glomerular filtration rate, CKD; chronic kidney disease.

### Observation of clinical event

Table 2 demonstrates the observational data. Total observation period for the primary endpoint reached 2467.3 patient-years, and that for the secondary endpoint was 2348.8 patient-years. Relevant bleeding from any site developed in 112 patients, with an incidence of 4.5 per 100 person-years. The breakdown was gastrointestinal bleeding (GIB) in 66 patients (2.7 per 100 person-years), intracranial bleeding in 9 patients (0.4 per 100 person-years), and bleeding from another site in 37 patients (1.5 per 100 person-years). Major adverse cardiac and cerebrovascular events (MACCE) developed in 110 patients, with an incidence of 4.7 per 100 person-years. Of all subjects, 15 patients dropped out of follow-up (1.4% of all subjects).

**Table 2:** Observational results from the study.

Observation period for primary endpoint	2467.3 patient-years
Observation period for secondary endpoint	2348.8 patient-years
**Outcome**	
All bleeding; n	112 (4.5 per 100 person-years)
Gastrointestinal tract	66 (2.7 per 100 person-years)
Upper	20
Middle	1
Lower	45
Intracranial	9 (0.4 per 100 person-years)
Others	37 (1.5 per 100 person-years)
Cutaneous/Subcutaneous	10
Nasal cavity	9
Urinary tract	8
Oral cavity	5
Ocular region	4
Joint	1
MACCE; n	110 (4.7 per 100 person-years)
Cardiac death	16
Myocardial infarction	5
Admission due to exacerbation of heart failure	65
Systemic thrombosis	24
Dropout; n	15 (1.4%)

MACCE; major cardiac or cerebrovascular events.

### Uni- and multivariate analyses

Table 3 shows the results of uni- and multivariate analyses regarding associations between bleeding and clinical factors at baseline. Development of all bleeding events correlated positively with very elderly status, CKD, LDA and NSAIDs. Regarding GIB, development of bleeding was significantly associated with the same factors, including very elderly status, along with advanced malignancies and history of GIB (Table 4).

**Table 3:** Clinical factors at baseline associated with incidence of all bleeding events in patients on direct oral anticoagulants.

Characteristics	Crude HR (95%CI)	*p* Value	Adjusted HR (95%CI)	*p* Value
Very elderly (≥ 85) (n; 112)	**2.163 (1.302–3.594)**	**0.002**	**2.15 (1.29–3.58)**	**0.003**
Sex (male)	0.840 (0.572–1.232)	0.372		
Low BW (< 40 kg)	0.769 (0.244–2.424)	0.654		
DOAC overdose	1.609 (0.593–4.366)	0.351		
**Comorbidities**				
Hypertension	1.263 (0.721–2.213)	0.415		
DM	0.932 (0.608–1.429)	0.748		
Dyslipidemia	1.417 (0.974–2.061)	0.068		
CHF	1.290 (0.875–1.903)	0.199		
IHD	1.245 (0.833–1.863)	0.285		
CVD	0.675 (0.371–1.229)	0.198		
PAD	0.898 (0.394–2.045)	0.798		
COPD	0.967 (0.471–1.985)	0.926		
CKD	**2.633 (1.283–5.406)**	**0.008**	**2.482 (1.200–5.134)**	**0.014**
Malignancy	1.660 (0.962–2.862)	0.068		
Past GIB	2.268 (0.835–6.158)	0.108		
**Medications**				
LDA	**1.527 (1.014–2.300)**	**0.043**	**1.522 (1.008–2.297)**	**0.046**
P2Y12	1.252 (0.746–2.102)	0.395		
NSAIDs	**3.070 (1.550–6.079)**	**0.001**	**3.303 (1.665–6.554)**	**0.001**
Steroids	0.910 (0.371–2.233)	0.837		
PPIs	0.773 (0.535–1.118)	0.171		

The crude and adjusted hazard ratios were evaluated by Cox proportional hazard analysis with stepwise methods. HR; hazard ratio, CI; confidence interval, BW; body weight, DOAC; direct oral anticoagulants, DM; diabetes mellitus, CHF; chronic heart failure, IHD, ischemic heart disease, CVD; cerebrovascular disease, PAD; peripheral artery disease, COPD; chronic obstructive pulmonary disease, CKD; chronic kidney disease, GIB; gastrointestinal bleeding, LDA; low dose aspirin, P2Y12; adenosine diphosphate receptor P2Y12 antagonists, NSAIDs; nonsteroidal anti-inflammatory drugs, PPIs; proton pump inhibitors. Values of *p* < 0.05 were regarded as significant.

**Table 4:** Clinical factors at baseline associated with incidence of gastrointestinal bleeding in patients on direct oral anticoagulants.

Characteristics	Crude HR (95%CI)	*p* Value	Adjusted HR (95%CI)	*p* Value
Very elderly	**2.258 (1.177–4.334)**	**0.014**	**2.256 (1.165–4.366)**	**0.016**
Sex (male)	0.787 (0.478–1.294)	0.345		
Low BW (< 40 kg)	1.341 (0.421–4.275)	0.620		
DOAC overdose	1.378 (0.337–5.632)	0.671		
**Comorbidities**				
Hypertension	1.136 (0.561–2.298)	0.723		
DM	0.979 (0.564–1.700)	0.940		
Dyslipidemia	1.635 (0.993–2.692)	0.053		
CHF	1.047 (0.638–1.717)	0.856		
IHD	1.442 (0.864–2.407)	0.162		
CVD	1.158 (0.605–2.215)	0.658		
PAD	0.765 (0.240–2.437)	0.650		
COPD	0.597 (0.187–1.903)	0.383		
CKD	**2.849 (1.144–7.096)**	**0.025**	**2.499 (0.988–6.320)**	**0.053**
Malignancy	**2.389 (1.274–4.478)**	**0.007**	**2.340 (1.243–4.403)**	**0.0089**
Past GIB	**3.901 (1.417–10.742)**	**0.008**	**3.109 (1.106–8.734)**	**0.031**
**Medications**				
LDA	**1.937 (1.161–3.232)**	**0.011**	**2.124 (1.249–3.540)**	**0.005**
P2Y12	1.552 (0.828–2.911)	0.170		
NSAIDs	**4.806 (2.287–10.101)**	**< 0.001**	**4.624 (2.169–9.858)**	**< 0.001**
Steroids	0.941 (0.295–2.998)	0.918		
PPIs	0.875 (0.539–1.418)	0.587		

The crude and adjusted hazard ratios were evaluated by Cox proportional hazard analysis with stepwise methods. HR; hazard ratio, CI; confidence interval, BW; body weight, DOAC; direct oral anticoagulants, DM; diabetes mellitus, CHF; chronic heart failure, IHD, ischemic heart disease, CVD; cerebrovascular disease, PAD; peripheral artery disease, COPD; chronic obstructive pulmonary disease, CKD; chronic kidney disease, GIB; gastrointestinal bleeding, LDA; low dose aspirin, P2Y12; adenosine diphosphate receptor P2Y12 antagonists, NSAIDs; nonsteroidal anti-inflammatory drugs, PPIs; proton pump inhibitors. Values of *p* < 0.05 were regarded as significant.

Table 5 presents analytical results concerning MACCE. Positive factors included CHF, peripheral arterial disease (PAD), and NSAIDs.

**Table 5:** Clinical factors at baseline associated with incidence of major cardiac or cerebrovascular events in patients on direct oral anticoagulants.

Characteristics	Crude HR (95%CI)	*p* Value	Adjusted HR (95%CI)	*p* Value
Very elderly	1.335 (0.713–2.499)	0.366		
Sex (male)	1.025 (0.686–1.531)	0.904		
Low BW (< 40 kg)	1.068 (0.393–2.898)	0.693		
DOAC underdose	0.899 (0.593–1.361)	0.614		
**Comorbidities**				
Hypertension	**2.037 (1.029–4.032)**	**0.041**		
DM	0.965 (0.628–1.482)	0.870		
Dyslipidemia	1.298 (0.890–1.891)	0.145		
CHF	**2.245 (1.445–3.487)**	**< 0.001**	**2.243 (1.444–3.483)**	**< 0.001**
IHD	**1.450 (0.973–2.162)**	**0.034**		
CVD	1.430 (0.896–2.284)	0.134		
PAD	**2.383 (1.359–4.176)**	**0.002**	**2.457 (1.401–4.311)**	**0.0024**
COPD	1.413 (0.758–2.635)	0.277		
CKD	2.068 (0.908–4.712)	0.084		
Malignancy	0.620 (0.272–1.412)	0.255		
Past GIB	0.564 (0.079–4.042)	0.568		
**Medications**				
LDA	**1.727 (1.152–2.588)**	**0.008**		
P2Y12	1.079 (0.614–1.896)	0.791		
NSAIDs	**2.504 (1.161–5.400)**	**0.019**	**2.534 (1.173–5.475)**	**0.0184**
Steroids	0.693 (0.255–1.881)	0.494		
PPIs	1.262 (0.861–1.848)	0.233		

The crude and adjusted hazard ratios were evaluated by Cox proportional hazard analysis with stepwise methods. HR; hazard ratio, CI; confidence interval, BW; body weight, DOAC; direct oral anticoagulants, DM; diabetes mellitus, CHF; chronic heart failure, IHD, ischemic heart disease, CVD; cerebrovascular disease, PAD; peripheral artery disease, COPD; chronic obstructive pulmonary disease, CKD; chronic kidney disease, GIB; gastrointestinal bleeding, LDA; low dose aspirin, P2Y12; adenosine diphosphate receptor P2Y12 antagonists, NSAIDs; nonsteroidal anti-inflammatory drugs, PPIs; proton pump inhibitors. Values of *p* < 0.05 were regarded as significant.

### Characteristics of very elderly patients

Table 6 summarizes differences of outcomes between very elderly patients ≥ 85 years old and younger patients. The incidence of MACCE in very elderly patients was similar to that in younger patients. On the other hand, the incidence of bleeding among very elderly patients was much higher than that among younger patients. In particular, the incidence of GIB in very elderly patients was 5.9 per 100 person-years, compared to 2.4 per 100 person-years in younger patients. Intracranial bleeding developed at a higher rate in very elderly patients than in younger patients, although the number of patients investigated was small (Table 6).

**Table 6:** Difference in incidence of clinical events between very elderly and younger patients during direct oral anticoagulant therapy.

Characteristics	Younger patients (< 85, n = 974)	Very elderly patients (≥ 85, n = 112)	*p* Value*
Observation period for bleeding (person-years)	2281.3	186	
Observation period for MACCE (person-years)	2170.2	178.6	
All bleeding (n = 112) (per 100 person-years)	94 (4.1)	18 (10.1)	< 0.01
Gastrointestinal (n = 66) (per 100 person-years)	55 (2.4)	11 (5.9)	0.01
Intracranial (n = 9) (per 100 person-years)	5 (0.2)	4 (2.2)	< 0.01
Others (n = 37) (per 100 person-years)	34 (1.5)	3 (1.6)	0.933
MACCE	99 (4.6)	11 (6.2)	0.363
Fatal bleeding	1	2	0.15

*Statistical evaluation was made using log-rank test. Abbreviations: MACCE; major adverse cardiac and cerebrovascular event.

## Discussion

The present study indicated that initiation of DOAC for very elderly AF patients ≥ 85 years old represented a significant risk factor for hemorrhage during treatment. The incidence of all bleeding (4.5% per 100 person-years) seems comparable to that from a randomized controlled trial where the incidence of major bleeding in patients ≥ 75 years old ranged from 3.3 per 100 person-years to 5.1 per 100 person-years^6–9^. When limited to very elderly patients ≥ 85 years old, however, the incidence per year reached 10%, while that of younger patients remained at 4.1% (Table 6). Conversely, occurrence of MACCE showed little difference between patient groups. In comparison with previous data that showed an age-related increase in cerebral thrombosis in patients with AF when anticoagulants were not administered, the present results imply that DOACs successfully suppressed the development of thrombosis among the very elderly^16–19,21–23^. According to a prospective cohort study that enrolled 464 patients on DOACs initiated at ≥ 85 years old, the incidences of GIB and thrombotic events were 2.00 per 100 person-years and 1.84 per 100 person-years, respectively^24^. The main reason for the higher incidence of GIB in the present study (5.9 per 100 person-years) was presumably the higher prevalence of the use of antiplatelet agents. LDA and adenosine 2 phosphate receptor P2Y12 antagonists were prescribed at rates of 20.2% and 13.1%, respectively, in our study. On the other hand, the prevalence of antiplatelet drugs was only 6.5% in the aforementioned study^24^. Regarding the incidence of thrombotic events, direct comparison of our results with that study is difficult, because the endpoints of each study differed. We adopted MACCE, while Poli et al. chose systemic thrombosis. The higher prevalence of heart failure in our study might be attributable to this discrepancy. In any case, systemic embolism is often serious and accompanied by sequelae, and we basically agree with the opinion that appropriate administration of DOACs should be considered even in the very elderly, regardless of the increased risk of bleeding. However, physicians should pay greater attention to hemorrhagic complications, particularly GIB, when initiation of DOACs is planned for patients ≥ 85 years old.

The gastrointestinal tract was the most common site of bleeding, with an incidence of 2.7 per 100 person-years; this seems comparable with results from randomized trials, where the incidence of major GIB ranged from 1 to 3 per 100 person-years^6–9,25,26^. Risk factors for GIB resembled those for all bleeding in the present study. On multivariate analysis, very elderly patients showed a significantly higher risk of GIB, with an adjusted hazard ratio of 2.256. Other factors included LDA, NSAIDs, CKD, malignancy, and history of GIB. HAS-BLED score, which shows risk factors for bleeding during warfarin treatment, indicates a past history of bleeding as an obvious risk factor for future bleeding. What is known about the risk of gastrointestinal bleeding with DOAC therapy is that past GIB was a significant risk. Sengupta et al. reported that at 90 days after discharge from hospitalization for initial GIB, 3.6% of patients who resumed DOACs were readmitted with recurrent GIB^27^. The incidence of GIB was much higher than reported in randomized trials, suggesting that a past history of GIB is strongly associated with GIB even among patients receiving DOACs. To reduce troublesome GIB, meaning a reduction of all bleeding events, avoidance of other risks is desirable; for example, the necessity for LDA or NSAIDs should be carefully reassessed, particularly in patients with CKD, history of GIB, or advanced malignancies. A previous report indicated PPIs as significant suppressors of GIB during DOAC therapy^28^, but this was not a significant factor underlying GIB in this study. That might be because the main site of GIB was the lower GI tract, rather than the upper GI tract in the present study. In our previous study, development of upper GIB was suppressed by PPI, whereas lower GIB was unaffected by PPI^29^.

Other factors significantly associated with bleeding included CKD, LDA, and NSAIDs. Concomitant administration of LDA or NSAIDs was related to not only bleeding but also MACCE. Those risks of bleeding, particularly GIB, are well known, with the mechanism of mucosal injury being the suppression of prostaglandin^30,31^. However, the association with MACCE remains unclear. CKD was also a factor related to bleeding. Although data about DOACs remain limited, CKD is regarded as a risk factor for both thrombosis and bleeding in patients with AF, which seems similar to our result^32–35^.

Clinical factors associated with MACCE differed from those associated with bleeding. Known risk factors for thrombosis in AF patients included CHADS2 and CHA2DS2-VASc scores, CHF, hypertension, age, diabetes mellitus, previous stroke or ischemic attack, and vascular disease. These have been used and validated as optimal in AF patients without anticoagulants, although their applicability remains uncertain in patients on DOACs^12^. In the present study, CHF and PAD showed significant associations with MACCE, both of which are components of CHA2DS2-VASc score.

Limitations to this study included the single facility, the retrospective study design, and the small sample size. In particular, since only 112 patients were ≥ 85 years old, the present results should be interpreted with care. In retrospective studies, the number of dropouts might often be a problem, but remained at 1.4% in this study, and was thus considered unlikely to have exerted any substantial effect on the results. Research in multiple facilities is desirable in the future.

In conclusion, the present study showed that bleeding was common along with thrombotic events among patients taking DOACs. The most common bleeding event was GIB. Some clinical factors including very elderly status, CKD, and concomitant use of LDA and NSAIDs were significantly associated with bleeding during DOAC administration. Regarding GIB, additional coexistence of malignancy and history of GIB showed positive correlations. When initiation of DOACs is considered among very elderly patients, risk of bleeding, and GIB, in particular, should be fully assessed.

## Methods

### Study subjects

**Figure 1:**
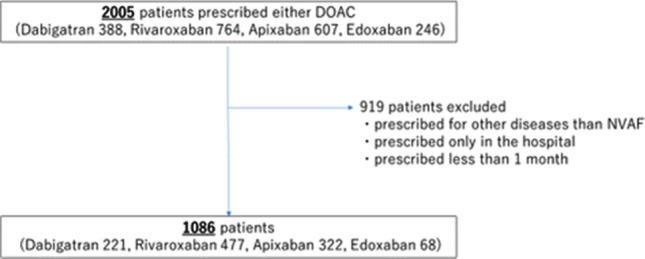
Flowchart for selection of study subjects.

Participants in this study were selected from patients at a single institution in Tokyo, Japan. All 2005 patients who had been prescribed a DOAC (dabigatran, rivaroxaban, apixaban, or edoxaban) between April 2011 and June 2017 were identified from prescription lists. Patients given DOACs for diseases other than non-valvular AF, prescribed DOACs only in hospital, or given DOACs for < 1 month were excluded. As a result, 1086 patients in total were enrolled as study subjects (Fig. 1). Primary endpoints were clinically relevant bleeding (Bleeding Academic Research Consortium (BARC) type 2–5), or discontinuation of prescription^20^. BARC proposes 5 bleeding types. Type 0 is no bleeding. Type 1 is bleeding that is not actionable and does not cause the patient to seek medical attention. Type 2 bleeding includes any clinically overt sign of hemorrhage that is actionable and requires diagnostic studies, hospitalization, or treatment by a healthcare professional. Type 3 bleeding is divided into 3 categories. Type 3a bleeding includes any transfusion with overt bleeding plus a hemoglobin drop of 3 to < 5 g/dL (provided the hemoglobin drop is related to bleeding). Type 3b bleeding includes overt bleeding plus a hemoglobin drop of ≥ 5 g/dL (provided the hemoglobin drop is related to bleeding), cardiac tamponade, bleeding requiring surgical intervention for control (excluding dental/nasal/skin/hemorrhoid), and bleeding requiring intravenous vasoactive agents. Type 3c bleeding includes intracranial hemorrhage and intraocular bleeding compromising vision. Type 4 bleeding is associated with procedures of coronary artery bypass grafting, such as perioperative intracranial bleeding within 48 h and reoperation after closure of sternotomy for the purpose of controlling bleeding. Type 5 bleeding is fatal. Secondary endpoints were development of MACCE including cardiac death, myocardial infarction, exacerbation of heart failure, and systemic thrombosis.

### Data collection

All data were collected from the medical records of subjects. The clinical course was reviewed every month until the end of December 2018, and observations ceased when the patient reached an endpoint or stopped visiting our institution for > 6 consecutive months without documented reason (regarded as “dropout” cases). The site of bleeding was identified where possible. Baseline characteristics of subjects at the time of DOAC initiation were also investigated, including biographic data (age, sex, height, and weight), type of DOAC and initial dose, comorbidities (hypertension, dyslipidemia, diabetes mellitus, CHF, ischemic heart disease, cerebrovascular disease, PAD, CKD, chronic obstructive pulmonary disease, liver cirrhosis, and advanced malignant diseases), history of GIB, and concomitant medications (steroids, NSAIDs, LDA, adenosine diphosphate receptor P2Y12 antagonist, or PPI). Uni- and multivariate analyses were used to clarify significant relationships between development of relevant bleeding or MACCE and clinical factors. We also focused on very elderly patients ≥ 85 years old, to estimate impacts on events compared with the younger patient group.

### Statistics

All statistical analyses were performed using SPSS Statistics version 24 software (IBM Japan, Tokyo, Japan). Differences in ratios or values between groups were evaluated using the chi-square test or Student’s t-test. Cox proportional hazard analysis with the stepwise forward likelihood method was used in uni- and multivariate analyses, to clarify significant clinical factors related to the development of bleeding or thrombotic events. The criterion for selecting covariates for multivariate analyses was pre-specified as a value of *p* < 0.1 in univariate analysis. Kaplan–Meier curves were adapted to show differences in the incidence of events between groups, where significance was evaluated using log-rank testing. Values of *p* < 0.05 were regarded as significant.

### Ethics

This protocol was approved by the institutional review board at Teikyo University prior to the study (TU19-140). All methods were carried out in accordance with relevant guidelines and regulations. The need to obtain informed consent was waived by the ethics committee that approved the study, given the retrospective design of the study.

## Data Availability

The data supporting the findings of this study are available on request from the corresponding author, K.A.; abe@med.teikyo-u.ac.jp at Teikyo University School of Medicine. These data are not publicly available, as they contain information that could compromise the privacy of research participants.
